# Secondary Schizophrenia

**DOI:** 10.1371/journal.pmed.0020279

**Published:** 2005-09-27

**Authors:** Dave Hambidge

**Affiliations:** **1**StaffordshireUnited Kingdom

May I respectfully highlight a potential confounding factor to interpreting an otherwise excellent and provoking study by Saha et al. [1].

In their recent overview of secondary schizophrenia (defined as “a disparate range of brain disorders that can, uncommonly, give rise to schizophrenia like symptomology” [2]), Hyde and Lewis [2] concluded that, overall, there was a prevalence rate of 5%–8% for psychoses of likely identifiable organic etiology amongst a series of relatively unselected patients. They suggest screening procedures in new cases of psychosis, including schizophrenia, with a battery of blood tests, a urine drug screen (UDS), and an electroencephalogram (EEG) as first-line investigations.

Between September 2000 and November 2003, I interviewed and studied the medical records of 56 patients in northwest England, who were appealing against detention under the Mental Health Act (1983), and who had been admitted for the first time within the last ten years [3]. They were all referred to me by their solicitors to prepare Legal Aid/Legal Services Commission–funded independent reports for their Mental Health Review Tribunal hearings. For each patient, I recorded which of the organic investigations suggested by Hyde and Lewis, if any, had been undertaken.

Of the 56 patients, ten were being detained for the first time (three females and seven males, detained on average for 39 weeks) and 13 had been detained for over one year (two females and 11 males, detained on average for 106 weeks). Whilst all except two of the 56 patients had some combination of blood tests recorded, 55% did not have a UDS and 83% did not have an EEG. Syphilis serology was examined for in only two patients of the latter group and none of the former. Therefore, my findings suggest that secondary schizophrenias may not be investigated for in most detained patients with a schizophrenia-like illness in England.

As secondary schizophrenias are present in 5%–8% of such cases, some of the variability in rates found by these authors must be related to the differing diagnostic rigour used to exclude secondary causes.
